# Peptide ligand screening of α-synuclein aggregation modulators by *in silico *panning

**DOI:** 10.1186/1471-2105-8-451

**Published:** 2007-11-16

**Authors:** Koichi Abe, Natsuki Kobayashi, Koji Sode, Kazunori Ikebukuro

**Affiliations:** 1 Department of Biotechnology, Tokyo University of Agriculture and Technology, 2-24-13 Naka-cho, Koganei-shi, Tokyo, 184-8588, Japan

## Abstract

**Background:**

α-Synuclein is a Parkinson's-disease-related protein. It forms aggregates *in vivo*, and these aggregates cause cell cytotoxicity. Aggregation inhibitors are expected to reduce α-synuclein cytotoxicity, and an aggregation accelerator has recently been reported to reduce α-synuclein cytotoxicity. Therefore, amyloid aggregation modulating ligands are expected to serve as therapeutic medicines.

**Results:**

We screened peptide ligands against α-synuclein by *in silico *panning, a method which we have proposed previously. In this study, we selected as the target a very hydrophobic region known as the amyloid-core-forming region. Since this region cannot be dissolved in water, it is difficult to carry out the *in vitro *screening of its peptide ligand. We carried out 6 rounds of *in silico *panning using a genetic algorithm and a docking simulation. After the *in silico *panning, we evaluated the top peptides screened *in silico *by *in vitro *assay. These peptides were capable of binding to α-synuclein.

**Conclusion:**

We demonstrated that it is possible to screen α-synuclein-binding peptides by *in silico *panning. The screened peptides bind to α-synuclein, thus affecting the aggregation of α-synuclein.

## Background

Protein misfolding and aggregation are involved in many diseases, such as Alzheimer's disease, Parkinson's disease and Prion disease, and such proteins accumulate as inclusion bodies in the brain. Lewy bodies are inclusion bodies observed in Parkinson's-disease patients. The major component of the Lewy body is amyloid-like fibrils of α-synuclein [1]. The familial mutants of α-synuclein A53T, A30P and E46K accelerate α-synuclein aggregation and/or fibrillation and cause autosomal-dominant Parkinson's disease [2-4]. These results strongly support the idea that α-synuclein is the pathogenic protein of Parkinson's disease. It is known that α-synuclein is one of the natively unfolded proteins which have little or no ordered secondary structure under physiological condition. However, changes in various environmental factors (*e.g*., pH, ion strength, agitation) induce the formation of α-synuclein aggregates and amyloid-like fibrils *in vitro *[5]. Especially, the aggregates called "protofibrils," an intermediate in the fibrillogenesis process, have more cytotoxicity than the amyloid-like fibrils of most of the proteins which generate fibrils [6]. Therefore, aggregation inhibitors are expected to serve as therapeutic medicines, and their effect against several amyloid-forming proteins has been reported [7-11]. However, inhibitors which do not inhibit protofibril formation but amyloid fibril formation, such as L-DOPA, enhance cytotoxicity [12]. Recently, Bodner *et al*. have reported that aggregation accelerator compounds decrease α-synuclein cytotoxicity [13]. Thus, aggregation accelerators are also expected to serve as therapeutic medicines.

To obtain an effective peptidic aggregation inhibitor, the peptide should bind to the region that plays an important role in amyloid fibril formation. The hydrophobic central region of α-synuclein called the "non-amyloid-β component of the amyloid plaque" (NAC) (residues 61–95) is the most important region in the formation of amyloid-like fibrils. In particular, some of the partial peptides in NAC, such as α-synuclein 68–78 (GAVVTGVTAVA), form amyloid-like fibrils by themselves [14]. We have also reported that a double mutant (V70T and V71T) of α-synuclein does not exhibit aggregation and fibrillation activity. Thus, the binding of peptide ligands to this region should affect α-synuclein aggregation. However, these amyloid-core-forming regions are so hydrophobic that these peptides cannot be dissolved in water. Therefore, computational screening would be useful to screen ligands against such a target.

**Figure 1:**
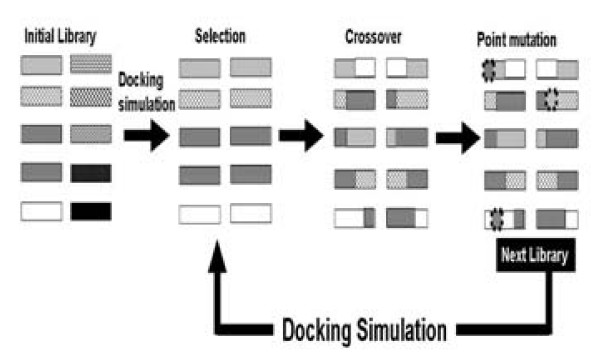
**Scheme of *in silico *panning**. *In silico *panning consists of two steps: a docking simulation and a genetic algorithm. The genetic algorithm consists of selection, crossover and point mutation. This figure shows one example of a genetic algorithm.

Since N-mer peptides have a huge sequence variety (20^n^), it is not sufficient to screen the huge sequence space randomly to obtain target peptides. In a previous study, we have reported an evolutional screening method using genetic algorithms (GAs) [15-18]. GAs are types of stochastic search algorithms that mimic Darwinian evolution. GAs can reduce the number of candidates which should be evaluated, and they have been effectively applied to screenings of binding poses for docking simulations [19] and the optimization of lead compounds [20,21]; these studies make reference to our reports. In such screenings, the peptide sequence can be optimized by evaluating the biological or chemical activity. Previously, we have proposed a peptide screening method using GAs combined with docking simulations: *in silico *panning [22]. The scheme of *in silico *panning is shown in Figure 1. The first step is the random design of the initial generation. The second step is the calculation of the docking energy between the target and the peptides, and the selection of the superior peptides. The third step is the crossover of the superior peptide sequences and the introduction of mutations into these sequences; the mutated sequences are then used for the next round and the operation is repeated. We have already succeeded in obtaining peptide inhibitors for glucose dehydrogenase using this method [22].

In related studies, ADAPT and ENPDA have been reported [23,24]. ADAPT is a program for designing small molecules, and ENPDA is a program for designing peptide ligands that is similar in methodology to our work; both methods were effective in screening ligands. In this study, we carried out *in silico *panning in order to screen the peptides which bind to the α-synuclein's amyloidogenic region and affect α-synuclein fibril formation.

## Results

### *In silico *panning

We selected an 11-mer peptide, α-synuclein 68–78 (GAVVTGVTAVA), as the target region. Although the three-dimensional structure of α-synuclein has been reported (1XQ8 [25]), this structure is in the Sodium Dodecyl Sulfate (SDS) micelle binding state and its major secondary structure is an α-helix. Under physiological condition, α-synuclein does not form a defined structure. Therefore, the unfolded state of the target 11-mer peptide is better suited as a docking target. Of course, docking simulations against unfolded proteins are challenging. However, amyloid fibrils generally have many β-sheet structures. Since the target region can aggregate by itself and mutants in this region lose their aggregation activity, this region is considered to form β-strands easily. In this study, we hypothesized that it would show a linear motif. We constructed the structure of the target 11-mer peptide using the MOE protein builder.

Since we have succeeded in screening peptide ligands by using a tetramer peptide in the previous study, we selected a tetramer peptide in this study as well. To carry out effective screening using a genetic algorithm, we restricted the type of amino acids composing the peptides. In this study, since the target region was very hydrophobic, we selected the hydrophobic amino acids and the polar amino acids that tended to form hydrogen bonds (glycine, alanine, valine, serine, threonine, proline, glutamine, asparagine, phenylalanine and tyrosine), and the total diversity is 10^4 ^= 10,000. However, even a docking simulation of all 10,000 sequences would be difficult because a peptide docking simulation requires more time than the docking simulation of a small compound. We therefore applied a genetic algorithm to the peptide *in silico *screening.

**Figure 2:**
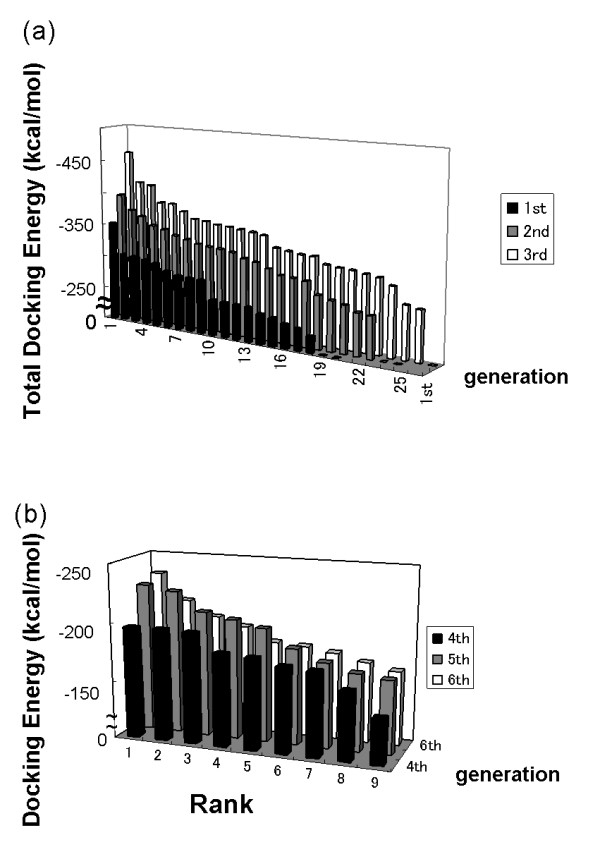
**Distribution of the docking energy**. The ranks of the peptide ligands in each generation are shown on the X- and Y-axes. The Z-axis shows the docking energies, which were calculated as follows: (a) docking total energy = electrostatic energies + van der Waals energies + conformational energy of the ligand; (b) interaction energy = total docking energy - conformational energy of the ligand. At the 1^st ^to the 3^rd ^generation, the peptides were evaluated by the total docking energy (a); at the 4^th ^to the 6^th ^generation, the peptides were evaluated by the interaction energy (b).

First, we evaluated the peptides by their docking energy: the sum of the intermolecular energy, the intramolecular energy of the ligand and the desolvation energy. Although the intramolecular energy is not related to the binding affinity, we included this energy to eliminate unstable ligands. From the 1^st ^to the 3^rd ^round, the docking energy decreased through *in silico *panning (Figure 2(a)). As expected, a progressive improvement of the docking energy was observed as the peptides evolved. However, since the intramolecular energy of some of the superior peptides was lower than their intermolecular energy, we removed the intramolecular energy of the ligands from the evaluation of the docking energy in the next round. We carried out a total 6 rounds. The results from the 4^th ^to the 6^th ^generation are shown Figure 2(b). The best peptide was obtained in the 6^th ^generation. Table 1 shows the peptide ranking after the 6^th ^generation. The peptides were ranked by their intermolecular energy. The top peptides had many glutamine residues that can form hydrogen bonds. Some peptides had a few hydrophobic residues, as calculated by Generalized Born/Surface Area in a continuous solvent model.

**Table 1:** Ranking of the superior peptides after *in silico *panning

**Total ranking**				
		**Energy(kcal/mol)**		
		
**No**	Sequence	**Total**	**Solvation**	**-ligand**

1	QSTQ	-390	-260	-233
2	GSQQ	-390	-260	-224
3	SQTQ	-393	-253	-221
4	AQTQ	-342	-251	-217
5	GSQN	-383	-243	-217
6	GQQV	-408	-255	-211
7	GTTP	115	-237	-211
8	ASTG	-329	-237	-210
9	GFNQ	-368	-237	-207
10	AGQV	-344	-237	-203

### Binding analysis by surface plasmon resonance

The affinities of the screened peptides were determined by surface plasmon resonance measurement. The screened peptides were immobilized on a CM5 sensor chip by amine coupling. In this study, we evaluated QSTQ, GSQQ, SQTQ and AQTQ which were the top peptides, and PTYF, which was an inferior peptide. The N-terminal glutamine could have formed pyroglutamine, and the N-terminal proline did not have a primary amine. Thus, we added an alanine residue to the N-terminals of these peptides to immobilize them on the CM5 sensor chip and evaluated them. The dissociation constants were determined using a Scatchard plot. We were able to confirm the peptide binding to α-synuclein, and the KD value of QSTQ was determined to be 19 μM. AQTQ also bound to α-synuclein with similar affinity of QSTQ. GSQQ and SQTQ have lower affinity than QSTQ and AQTQ. In the case of PTYF, its KD was more than 10 times that of QSTQ. Since docking simulation still has problems inaccuracy, we have to evaluate several peptides to find good ligands. In this study, we can obtain the high affinity peptide by assaying the top 4 peptides.

### Effect of synthetic peptide ligands on α-synuclein aggregation

**Figure 3:**
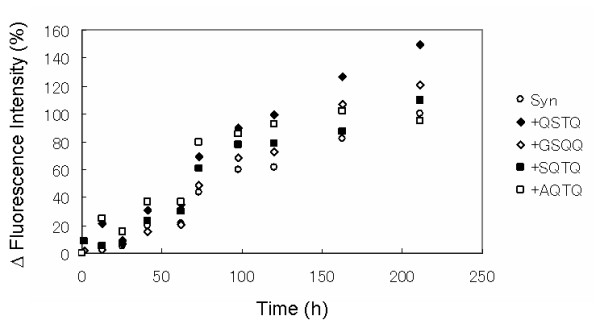
**Time course of α-synuclein fibril formation**. The Y-axis shows the normalized fluorescence intensity of Thioflavin T at 482 nm. 100% is the final fluorescence intensity of α-synuclein without peptide. The samples contained the following: α-synuclein (140 μM) without peptide, (Circle); QSTQ, (Black diamond); GSQQ, (Empty diamond); SQTQ (Black square); AQTQ, (Empty square). All peptide concentrations were 700 μM.

**Figure 4:**
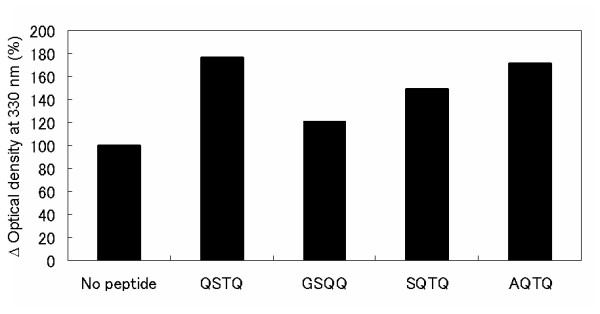
**Light scattering assay**. The Y-axis shows the normalized optical density at 330 nm. 100% is the optical density at 330 nm of α-synuclein without peptide.

To determine the effect of peptides on α-synuclein aggregation, we co-incubated α-synuclein with 5-fold molar excess of the screened peptides. The α-synuclein fibril amounts were evaluated by Thioflavin T (TfT) binding assay. It is known that when TfT binds to amyloid fibrils, the fluorescence intensity around 480 nm increases. The α-synuclein solution without peptide showed an increase in the fluorescence intensity of TfT after 40 h of shaking (Figure 3). On the other hand, the α-synuclein solutions with each top-ranking peptide showed a greater increase in the fluorescence intensities of TfT than that of the solution without peptide. Especially, the α-synuclein solution with the top peptide, QSTQ, showed a fluorescence intensity which was more than 1.5 times stronger than that of the solution without peptide. Next, to determine whether or not these increases in fluorescence intensity depend on the fibril amount, we examined the aggregation amount by a light scattering assay at 330 nm. The value of optical density at 330 nm of the α-synuclein solution with the top peptides was also higher compared with that of the α-synuclein solution without peptide, as shown in Figure 4. Especially QSTQ and AQTQ that have similar affinity showed optical density at 330 nm which was more than 1.5 times stronger than that of the solution with out peptide. The screened peptides showed the promotion of α-synuclein fibrillation.

## Discussion

Peptide docking simulation takes more time than the docking simulation of small molecules because of the structural flexibility of peptides. Therefore, it is difficult to evaluate the peptide library comprehensively by means of a docking simulation. Yoshimori *et al*. have proposed a stochastic approach to reduce the number of evaluated peptides [26]. Alternatively, we applied a GA to reduce the number of evaluated peptides. We call this method "*in silico *panning," and have previously reported the screening of a glucose dehydrogenase noncompetitive inhibitor. For the recognition of the target protein, peptides need to have functional amino acids, and the amino-acid sequence is more important than the position of each amino acid. In some cases, the combination of independent important amino acids results in loss of binding affinity. The GA can search the sequence space by optimizing the amino-acid sequence. The GA comprises three steps: selection, crossover and mutation. Through the operation of sequence crossover, it is possible to avoid falling into the local minimum. GAs are used in several fields, especially in informational science, and the results demonstrate the efficiency of GA-based screening. In this study, we evaluated 150 peptides *in silico*. This represents 1.5% of the total library, and we succeeded in obtaining the peptide that affects α-synuclein aggregation and fibrillation. Therefore, our approach was sufficiently effective for peptide ligand screening. At present, there are many docking simulation softwares, and some of them can perform the calculations more rapidly. Thus, *in silico *panning has the potential to be applied it to a larger library.

There are some reports of peptides that affect α-synuclein aggregation [7,27,28]. These peptides are homologous peptides. Amyloid-forming proteins, including α-synuclein, can form self-aggregates. Therefore, homologous peptides should bind to the proteins, and these peptides have the potential to act as protein aggregation inhibitors. It is known that peptides partially homologous to amyloid β bind to amyloid β and the modified homologous peptide inhibits amyloid β aggregation [9,10]. Concerning α-synuclein, El-Agnaf *et al*. have reported that peptides partially homologous in the NAC region, especially the α-synuclein 60–80 region, bind to α-synuclein across its full length, and that these peptides, modified to dissolve in water, inhibit α-synuclein aggregation[7]. Therefore, if the peptides binding these regions can be screened, they could be used to inhibit α-synuclein aggregation.

While there are many reports of aggregation inhibitors, there are some reports of aggregation accelerator peptides [7,27,28]. These are peptides homologous to the NAC region. Surprisingly, El-Agnaf *et al*. have reported that a peptide designed to inhibit α-synuclein aggregation actually accelerated the aggregation. This peptide lacks one amino acid from the α-synuclein aggregation inhibitor peptide sequence. The protein fibrillation process is assumed to comprise three steps: conformational change, the formation of an amyloid core through self-aggregation, and the elongation of amyloid fibrils. The amyloid-core-forming region is highly hydrophobic. Thus, this region should be isolated to some extent from the solvent. This region may become exposed to the solvent upon the conformational change resulting from the addition of certain factors, such as ligands, which might induce the aggregation of these regions.

The ligand with strong binding activity may inhibit aggregation, which is followed by conformational change. Since amyloid fibrils have a very stable conformation, it is thought that the binding ability of self-aggregation is very strong. Weakly bound ligands would not sufficiently inhibit this aggregation followed by conformational change. Therefore, peptides lacking some of the amino acids of the inhibitor peptide sequence would accelerate α-synuclein aggregation and induce fibrillation. In this study, the screened peptides may have bound to the hydrophobic core region, but the binding ability was not high. These peptides might induce conformational changes by binding to the hydrophobic region, and accelerate the α-synuclein aggregation and subsequent fibrillation.

## Conclusion

We demonstrated that *in silico *panning can be used to screen α-synuclein-binding peptides. Although α-synuclein does not have a defined structure, we were able to screen the peptides by hypothesizing that the target region forms a β-sheet structure. The screened peptides showed low dissociation constants (for example, *K*_D _= 19 μM) and affected α-synuclein aggregation and fibrillation. These peptides accelerated α-synuclein aggregation and fibrillation, and may be able to decrease α-synuclein cytotoxicity by decreasing the protofibril amount.

Since our *in silico *panning approach has a great potential and is applicable to a larger library, it may be useful for the ligand screening of certain disease-related proteins.

## Methods

### Construction of the initial generation and target

We designed the peptides of the initial generation randomly, without bias. Since the target peptide, α-synuclein 68–78, is very hydrophobic, we expected that the peptide ligands would bind to the target peptide through hydrophobic interaction and hydrogen bonding. To avoid an excessively large sequence space, we selected 10 amino acids: glycine, alanine, valine, serine, threonine, proline, glutamine, asparagine, phenylalanine and tyrosine. The molecular operating environment (MOE) from the Chemical Computing Group, Inc. was used for the model building. After the three-dimensional construction of these peptides, in order to optimize these peptides' local conformations, these structures were subjected to an MMFF94 energy minimization protocol until the root mean square of the conjugate gradient reached <0.05 kcal mol^-1 ^Å^-1^. The target peptide, α-synuclein 68–78, was constructed in the same way.

### Docking simulation

The docking simulation described here was carried out using the MOE-Dock default parameters based on the simulated annealing protocol. We selected the MMFF94s force field. The MOE suite utilized here includes an implemented version of the Generalized Born/Surface Area contact function that models the electrostatic contribution to the free energy of the solvation in a continuous solvent model. A docking box was constructed around α-synuclein 68–78. We carried out the MOE-Dock 30 run in our case.

### Genetic algorithm

We implemented a simple genetic algorithm to produce the next generation. After the docking simulation, the peptides were ranked by their calculated docking energy. The more or less superior peptide sequences were duplicated, and the sequences were recombined at a 1.0 crossover rate. Finally, single-amino-acid mutations were introduced into the peptides at a mutation rate of 5 to 20%. Population sizes of each generation are selected between 20 and 25 according to number of superior peptide.

### Expression and purification of α-synuclein

Human wild-type α-synuclein expressed in *Escherichia coli *BL21(DE3) cells transformed the pET28a/human α-synuclein WT plasmid. Following induction with 1 mM isopropyl-1-thio-β-D-galactopyranoside, the α-synuclein was purified by anion exchange chromatography [29]. The bacterial cells were harvested by centrifugation at 5,000 *g *for 10 min and lysed in 500 mM NaCl, 20 mM Tris-HCl, pH 8.0, and 1 mM EDTA. The lysate was then boiled for 20 min, and centrifuged for 10 min at 20,000 *g*. The supernatant was dialyzed against 20 mM Tris-HCl, pH 8.0, loaded onto a ResourseQ 6-ml column (Amersham Biosciences) equilibrated in the same buffer, and eluted with a 0 ~1 M NaCl gradient. The α-synuclein-containing fraction was dialyzed against 10 mM Tris-HCl, pH 7.4.

### Peptide synthesis and purification

All peptides were synthesized using 9-fluorenylmethyoxycarbonyl (Fmoc) chemistry and a solid phase system using the peptide synthesizer PSSM8 (Shimadzu Co., Ltd., Kyoto, Japan). The synthesis resin, TentaGel S PHB-coupled Fmoc-amino acid, was purchased from HiPep Laboratories. Deprotection/cleavage from the resin was performed through treatment with a 90:5:5 (v/v/v) trifluoroacetic acid (TFA)/water/triisopropylsilane mixture for 2 h. Since the glutamine at the N-terminal could undergo a side-reaction, we conducted the acetylation of the N-terminal amine. The synthesized peptides were purified by reverse-phase liquid chromatography using a C18 ODS-column (TOSOH). The mass weights were confirmed by ESI-MS (JEOL).

### Surface plasmon resonance

All peptides were immobilized onto the CM5 sensor chip by amine coupling. The CM5 sensor chip (Biacore) was activated by an equimolar mix of NHS (*N*-hydroxysuccinimide) and EDC (*N*-ethyl-*N*'-(dimethylaminopropyl) carbodiimide), coupled with 100 μg/mL of the synthesized peptide in pH-6.0 sodium acetate buffer, and then blocked with ethanolamine. For reference, only activation and protection was carried out, in the same way as mentioned above. The final coupling level of each peptide was approximately 100 RU. Each of the α-synuclein solutions was injected, and at different concentrations a signal increase was observed. The sensor surface was regenerated using 0.5% SDS. The dissociation constants were determined using a Scatchard plot and an equilibrium point.

### Fibril formation and analysis

All the protein samples were concentrated to approximately 4.0 mg/ml by means of Amicon Ultra-15 filters (Millipore) in 10 mM Tris-HCl, pH 7.4, and centrifuged at 150,000 *g *for 60 min to remove the insoluble aggregates. The peptides were precipitated in the same buffer and mixed with α-synuclein. The α-synuclein and peptide mixtures were incubated in 0.5 ml of the same buffer with 0.02% NaN_3 _at 37°C by shaking in 1.5 ml tubes. The protein concentrations were kept at approximately 2.0 mg/ml. Fibril formation was monitored by thioflavin T (TfT) fluorescence: 10 μl aliquots were sampled from the incubated samples and added to 1.0 ml of 25 μM TfT in 10 mM Tris buffer, pH 7.4. Fluorescence emission was measured at 485 nm at an excitation wavelength of 450 nm on a FP6500 spectrofluorometer (JASCO). The Rayleigh scattering at 330 nm was used to monitor the total aggregation.

## Authors' contributions

KA carried out the docking simulation, the determination of the peptide affinity against α-synuclein and the investigation of the effect of the peptides against α-synuclein aggregation and fibrillation. NK carried out the construction of the α-synuclein vector. KS and KI conceived the study, participated in its design and coordination, and helped to write the manuscript. All authors have read and approved the final manuscript.
